# Elevated Systemic L-Kynurenine/L-Tryptophan Ratio and Increased IL-1 Beta and Chemokine (CX3CL1, MCP-1) Proinflammatory Mediators in Patients with Long-Term Titanium Dental Implants

**DOI:** 10.3390/jcm8091368

**Published:** 2019-09-02

**Authors:** José Joaquín Merino, María Eugenia Cabaña-Muñoz, Adolfo Toledano Gasca, Alba Garcimartín, Juana Benedí, Fabio Camacho-Alonso, José María Parmigiani-Izquierdo

**Affiliations:** 1 Departamento de Farmacología, Farmacognosia y Botánica, Facultad de Farmacia, Universidad Complutense de Madrid (U.C.M), c/Plaza Ramón y Cajal s/n, 28040 Madrid, Spain; 2 Centro CIROM, Centro de Implantología y Rehabilitación Oral Multidisciplinaria, 30001 Murcia, Spain; 3 Department of Neuroanatomy, Instituto Cajal (CSIC), 28040 Madrid, Spain; 4 Faculty of Medicine, University of Murcia, 30100 Murcia, Spain

**Keywords:** amino acids, L-Kyn/L-Trp, His, kynurenine pathways, titanium dental implants and dental amalgam fillings, tryptophan (L-Trp), cytokines, IDO, chemokines, inflammation, cytokines, chemotaxis, CX3CL1 (fractalkine), melatonin, dentistry and medical toxicology

## Abstract

Titanium is the mean biocompatible metal found in dental titanium alloys (Ti-6Al-4V). The safety of certain dental biomaterial amalgams has been questioned in patients. The levels of several systemic cytokines (interleukin (IL)-1 beta, IL-4: pg/mL) and chemokines (monocyte chemoattractant protein-1 (MCP-1), soluble fractalkine (CX3CL1: pg/mL) were determined using ELISA and compared between these study groups. The study included 30 controls without dental materials (cont), 57 patients with long-term titanium dental implants plus amalgams (A + I group) as well as 55 patients with long-term dental amalgam alone (A group). All patients (except controls) have had dental titanium implants (Ti-6Al-4V) and/or amalgams for at least 10 years (average: 15 years). We evaluated whether systemic levels of cytokines/chemokines, kyn/L-trp ratio and aromatic amino acid levels (HPLC: mM/L, Phe, L-Trp, His, Treo) could be altered in patients with long-term dental titanium and/or amalgams. These systemic markers were evaluated in 142 patients. The A + I group had higher L-Kynurenine/L-Tryptophan ratios than patients with long-term dental amalgam fillings alone (A). In addition, levels of IL-1 Beta cytokine, CX3CL1 and MCP-1 chemokines were higher in the A + I group than in the A group (A). The increased L-kyn/L-trp ratio and MCP-1 and fractalkine receptor (CX3CR1) elevations could suggest enhanced chemotactic responses by these chemokines in the A + I group.

## 1. Introduction

The constituents of dental amalgams are mercury (Hg^++^: 50% by weight of the alloy powder), silver (Ag: 41% by weight), and tin (Sn), with copper (Cu^++^) and zinc (Zn^++^) as minority oligoelements (5–8%) [1,2,3]. These heavy metal/oligoelements are detected by Inductive Coupled Spectrometry (ICP-MS) in biological samples (urine, plasma or hair) [4] and amino acids can be detected by HPLC (High Chromatography Liquid) [5]. Several clinical studies have questioned the safety of dental biomaterials [6,7]. Prosthetic dental materials contain metals (Co, Cr, Au, Ag, Ti), resins, and ceramics, which, in combination, could provoke metal ion release through galvanism between metals [8]; this process may occur when implant alloys are close to dental amalgam fillings [9,10].

The biocompatible properties have made titanium alloys (Ti-6Al-4V) clinically important in oral surgery and in the orthopedics field [11]. Despite being considered biocompatible, some adverse reactions have been documented [12], including inflammation in vitro and allergy [13,14]. The safety of dioxide titanium nanoparticles (TiO_2_) has been questioned in several studies [14,15,16]. In fact, TiO_2_ toxicity was reported in human astrocytes in vitro [17].

The application of the cautionary principle for dental treatments is necessary and the use of safer protocols such as employing a nasal filter during dental amalgam removal could minimize heavy metal exposure in patients with dental biomaterials [2,7,11]. Understanding the possible mechanisms by which heavy metal release could induce inflammation could improve clinical treatment in medical toxicology and in the odontology field.

L-Tryptophan (L-Trp) is an aromatic amino acid that needs to be acquired through the diet and its deficiency can affect mood in patients [18,19]. Pineal cells take tryptophan up from the blood, and turn it into serotonin, which is converted into melatonin. Melatonin can induce beneficial effects on dental pathologies [20]. The proinflammatory interleukin (IL)-1 Beta cytokine has been implicated in implant failure [21].

A serotonin precursor, L-Trp, can also form melatonin as well as niacin (B3 vitamin). L-Trp deficiency reduces brain serotonin levels and lower Histidine (His) could decrease dopamine levels [22,23]. The activity of indoleamine 2,3-dioxygenase (IDO), a tryptophan-degrading enzyme, catabolizes L-Trp degradation to the L-Trp–kynurenine pathway and also forms other immunosuppressive metabolites [24]. The L-Trp conversion to kinurenine (Kyn), also known as the L-kyn/L-trp ratio, is an indirect index of indoleamine 2,3-dioxygenase enzymatic activity (IDO); IDO levels rise in inflammatory conditions and under stress [25,26].

Chemokines (chemotactic cytokines) play a major role in selectively recruiting monocytes, neutrophils and lymphocytes, as well as in inducing chemotaxis through the activation of G-protein-coupled chemokine receptors. The elevated expression of chemokines and their receptors can contribute to chronic inflammation. Monocyte chemoattractant protein-1 (MCP-1 = CCL2) is one of the key chemokines that regulate the migration and infiltration of monocytes/macrophages to inflammatory sites [27]. In addition, the soluble CX3CL1 (also called soluble fractalkine) chemokine ligand recognizes its delta CX3CR1 chemokine receptor; these delta chemokines are expressed by endothelial cells, monocytes, and lymphocytes among others. The CX3CL1 ligand is released by immune and non-immune cells in response to stress signals [28,29]. An analysis of these proinflammatory cytokines and chemokines could help evaluate inflammation levels in patients with long-term dental amalgam implants (Ti-6Al-4V) and amalgams.

### Aim

The present investigation examines the influence of long-term dental titanium implants on the L-Kyn/L-Trp ratio as well as whether changes in systemic inflammatory mediators (cytokines and CX3CL1 and MCP-1 chemokines) occur in patients with long-term dental titanium implants and/or dental amalgam fillings as compared with controls without dental biomaterials.

#### Secondary Aim

We have studied whether systemic amino acid levels (Trp, Treo, Phe, His) might be altered in patients with long-term titanium dental implants and amalgams (A + I) as compared to those with long-term dental amalgam fillings alone (at least for 10 years, average: 15) or controls without dental biomaterials

We consider whether higher systemic CX3CL1 (also termed soluble fractalkine), MCP-1 (CCL-2) and IL-1 Beta proinflammatory cytokine levels could predict or reflect systemic inflammatory response in patients with long-term dental titanium implants and amalgams (A + I) as compared to those with long-term dental amalgams alone (A) and controls without dental biomaterials.

## 2. Materials and Methods

### 2.1. Patients (Inclusion Criteria)

This study followed the tenets of the Declaration of Helsinki and was approved by the CIROM: (Centro de Implantología y Rehabilitación Oral Multidisciplinaria) ethical committee (#03-2016). All subjects were properly informed and signed the appropriate informed consent forms. All efforts have been made to protect patient privacy and anonymity. The CIROM Center has been approved and certified by AENOR: the Spanish Association for Standardization and Certification, Spain (CIROM Certificate for total dentist services; CD-2014-001 number; ER-0569/2014 following UNE-EN ISO 9001: 2015 update and UNE 179001-2011 Directive, Spain). All selected patients are aged between 44 and 72 years old (average: 55.5 years). The percentage of smokers is 9%, and their sociocultural status is medium-high (65% have at least a university degree). CIROM dentists revised 350 clinical histories before including potentially eligible patients. Finally, the present study enrolled 142 patients and the total number of excluded patients was 208. A total number of 46 patients did not meet the inclusion criteria because their dental titanium implants and amalgams are less than 10 years old. Finally, the implantologist (CIROM Clinic) selected a number of 142 patients, including control subjects (without dental materials); we determined the systemic L-kyn/L-trp amino acids levels as well as Phenyalanine (Phe), Tyrosine (Tyr), Treonine (Treo) and L-Tryptophan (Trp) levels by HPLC (mM/L: Indatxo, Basque Country, Spain) [5]. Last, levels of proinflammatory cytokines (IL-1 Beta, IL-4) and chemokines (soluble fractalkine (CX3CL1), MCP-1 = CCL-2) were quantified in trunk blood using ELISA (Leti duo set kits, Barcelona, Spain, *n* = 142).

The experimental design included three study groups: (a) patients who have had long-term dental titanium implants (Ti-6Al-4V) and amalgams for at least ten years (average: 15 years, Amalgam + Implant group, A + I, *n* = 57); (b) patients who have had long-term dental amalgams alone (Amalgam group, A, *n* = 55) for at least teen years (average: 15 years, A, *n* = 55); (c) healthy controls without dental biomaterials (Cont, *n* = 30). These control subjects had no metabolic or behavioral problems and had an average age of 44 years old; the average age for patients with long-term titanium and amalgams is 57 years old (A + I group) and patients with long-term dental amalgams alone have an average age of 46 years old (A group). We have selected patients who have had dental titanium implants and amalgams (A + I) for at least 10 years (average: 15 years), averaging four titanium implants (minimum: 1, maximum: 8) and four amalgams (minimum: 2, maximum: 6). Patients with long-term dental amalgam fillings alone also had an average of 4 fillings (minimum: 2, maximum: 6). The amalgam fillings were replaced by composites (bisphenol A free) following a safe protocol using nasal filters (^@^InspiraHealth, Spain) [30]. There are four quadrants in the mouth, and the amalgam fillings were progressively replaced by quadrant. The was no patient group with long-term titanium dental alloys alone because all the patients with implants had previously dental amalgam fillings.

**Figure 1:**
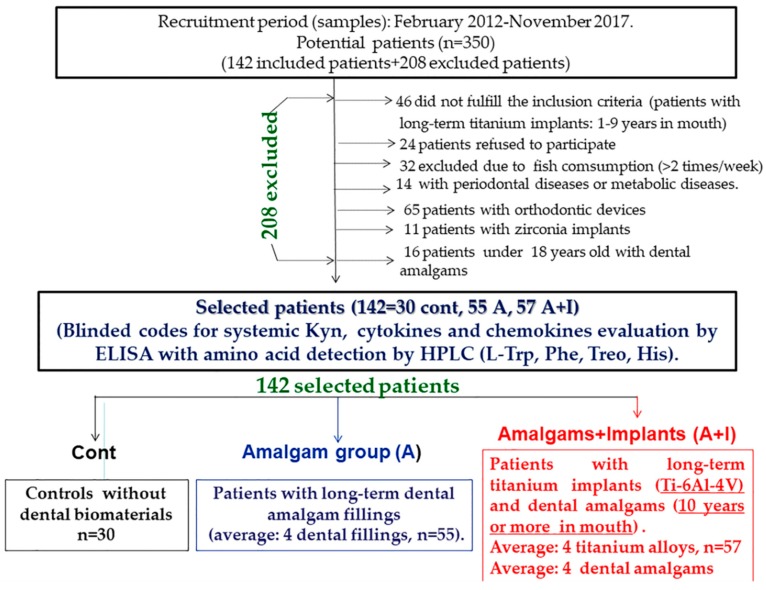
Study groups.

The patients’ economic and sociocultural status is medium/high; all live in Murcia (80%, 18% are males) and 20% are from Alicante (Spain, Europe). Patients live in Murcia (80%) and Alicante (20%, Spain, Europe); 18 percent of study patients are women (Figure 1).

### 2.2. Exclusion Criteria

Patients with a metabolic disease, diabetes, renal failure, gastrointestinal disease, a history of liver or kidney disease, lupus/autoimmune disease, or neurological/psychiatric disease (4th Edition, DSM IV), as well as any orthodontic device or periodontal disease were excluded from the study. Patients with hyperlipidemia were also not considered. A total number of 208 patients were excluded; 24 patients refused to participate and 32 were excluded due to their high fish consumption (more than twice a week). We discounted those with metabolic alterations or periodontal disease (*n* = 14) as determined by the periodontal Index of Community (WHO: World Health Organization, 1997, Federation Dentaire Internationale). Patients with orthodontic devices were excluded (*n* = 65) as were patients with zirconia implants (*n* = 11). Patients under 18 years old with dental amalgam fillings were excluded from this study (*n* = 16).

### 2.3. ELISA for Inflammatory Mediators (IL-1 Beta, IL-4, Macrophage Colony Protein-1: MCP-1 = CCL-2, and Soluble Fractalkin Levels CX3CL1 = sFK)

Il-1 Beta levels were measured following ELISA kit guidelines (Leti, Barcelona, Spain) and our own earlier protocol [31]. Samples were diluted (1:2 with BSA-phosphate) and a standard curve was made using different dilutions following the manufacturer’s instructions. All samples, including standards, were incubated overnight (o/n) in a humidified chamber; 50 μL of monoclonal IL-1 Beta antibody was added to the plate for 2 h at 37 °C. After 3 washes with washing buffer (phosphate-buffered saline PBS, containing 0.02% plus 0.02% Tween-20 and thrimerosal), 100 μL of polyclonal IL-1 Beta antibody was added to all wells for 1 h at room temperature (R.T). After 5 washes, 100 μL of a diluted horse-radish peroxidase (HRP)-conjugated anti-rabbit Ig G was added to the plate and incubated at room temperature (25 °C) for 30 min. After another wash with washing buffer, 100 μL of 0.002 mol/L ortho-phenylendiamine dihydrochloride (OPD) plus 3% hydrogen peroxide (H_2_O_2_) was added to citrate buffer in all plates for 10 min (R.T) following our laboratory protocol [31]. The colorimetric reaction was stopped by adding 50 μL of 2.5 N of sulfuric acid H_2_SO_4_ to the plate. The optical density (absorbance) was measured at 490 nm in triplicate (Microplate Reader spectrophotometer, Thermo Scientific). The amount of IL-1 Beta levels per sample were quantified by interpolation within the standard curve (STD); IL-4 and MCP-1 chemokines (also called CCL-2) were quantified with ELISA duo sets in plasma and expressed as pg/mL (ELISA duo set kit). These ELISA duo sets used a primary MCP-1 antibody according to the manufacturer’s instructions. The soluble fractalkine (CX3CL1) delta chemokine ligand was determined following the R&D ELISA kit (USA) system guidelines.

### 2.4. L-Kynurenine (L-Kyn)

Plasma L-Kynurenine (L-Kyn) levels were quantified following the manufacturer’s instructions (ELISA kit, IDK, Germany, K-7728); the detection range is between 1 and 10 μM L-Kyn without cross-reactivity with L-kynurenine analogs [L-Trp (0.01%), Hydroxil-DL-Trp (0.01%), Tyrosine, serotonin, Phenylalanine, L-Asn, and kynurenic acid]. The sensitivity for kynurenine is 100% [31].

### 2.5. Statistical Analysis

All data were analyzed using SPSS (v17.0), and Sigma Plot (11.0) software. The mean and S.E.M (Standard Media deviation) were evaluated for all estimated parameters; the S.E.M is the standard deviation divided by n root square, n being the sample size. Between-group comparisons were tested by Mann–Whitney in the absence of the homogeneity of variance. The ANOVA and post hoc Bonferroni analysis evaluated possible differences in the case of homogeneity of variance. Dunn’s task was applied for comparison in study groups for unequal sample size. A two-sided *p* value of *p* < 0.05 was considered to be statistically significant when *p* < 0.05, and highly significant if *p* < 0.01.

## 3. Results

### 3.1. Figure 2: Lower Systemic Trp Levels in Patients with Long-Term Dental Titanium Amalgams as Compared to Participants with Long-Term Dental Amalgams Alone

**Figure 2:**
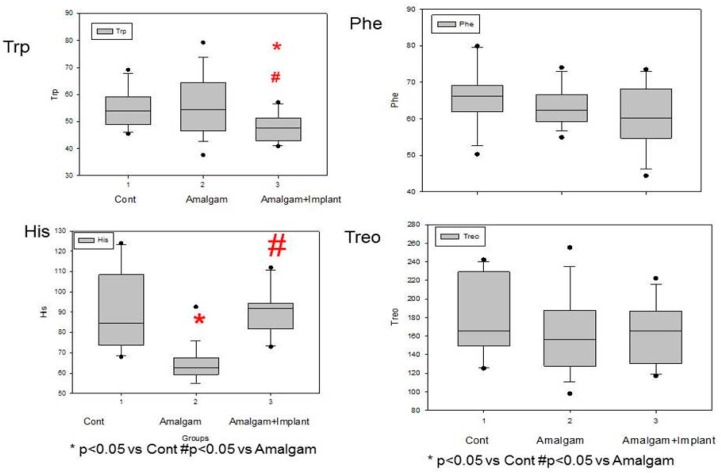
Lower systemic Trp or His levels in patients who have long-term titanium dental implants and amalgams (for at least 10 years: average: 15, A + I) than in participants with long-term dental amalgams alone (A). **Cont:** control (*n* = 30). **Amalgam** (A, *n* = 55). **Dental titanium implants + Amalgam** (A + I, *n* = 57). * *p* < 0.05 vs. Control group (Cont), ^#^ *p* < 0.05 vs. Amalgam group (A).

Figure 2 showed lower systemic tryptophan (L-Trp) levels among patients with long-term dental titanium implants and amalgams than in controls (*p* < 0.05). We observed lower systemic L-Trp levels in the A+I group than in the A group (*p* < 0.05). However, L-Trp levels did not differ between patients with long-term amalgams alone and healthy controls (*p* > 0.05; n.s). Other aromatic amino acids like Phe or Treo were not significantly different between patients with long-term titanium dental implants and amalgams than healthy controls, respectively (*p* > 0.05; n.s).

### 3.2. Patients Who Had Long-Term Dental Amalgams Have Lower Systemic His Levels Than Control Subjects

Patients with long-term amalgams alone (A group) have lower systemic Histidine levels (His, *p* < 0.05) than participants with long-term titanium dental implants and amalgams (A + I group). Patients with long-term dental amalgams alone have significantly lower systemic His levels than controls (*p* < 0.05, see Figure 2). There were no different effects on Treo and Phe levels between the groups (*p* > 0.05, n.s, in all cases).

### 3.3. L-Kynurenine

**Figure 3:**
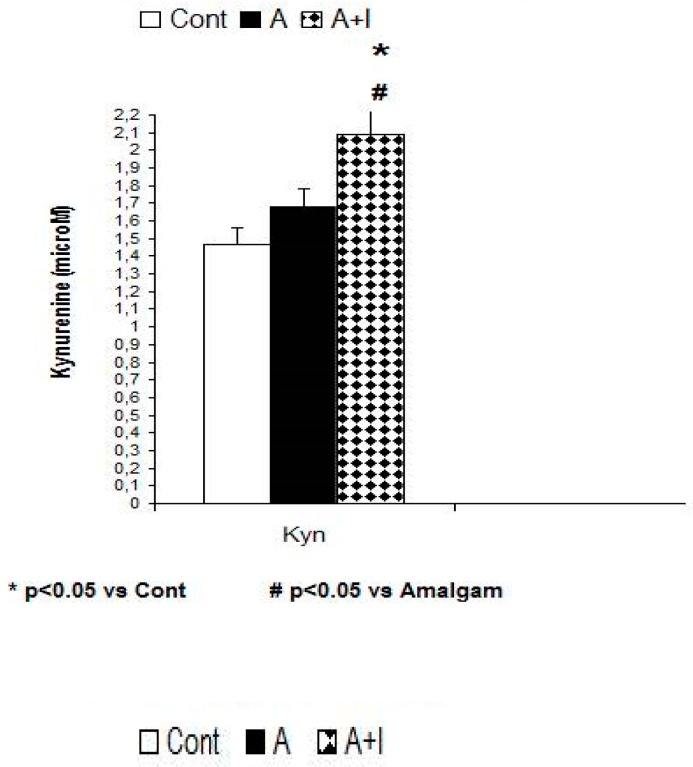
Increased systemic Kyn levels in patients with long-term titanium implants and amalgams (A + I). **Cont:** control (*n* = 30). **Amalgam** (A, *n* = 55). **Dental titanium implants + Amalgam** (A + I, *n* = 57). * *p* < 0.05 vs. Control group (Cont), ^#^ *p* < 0.05 vs. Amalgam group (A).

Systemic kyneurine levels were higher among patients with long-term dental titanium implants and amalgams than in participants with long-term dental amalgams alone [F (2, 140) = 5.96, *p* = 0.035, *p* < 0.05] and were also higher than in healthy controls (*p* < 0,05, see Figure 3 and Table 1).

### 3.4. Ratio L-Kynurenine/L-Trp: An Index of IDO Activity

**Figure 4:**
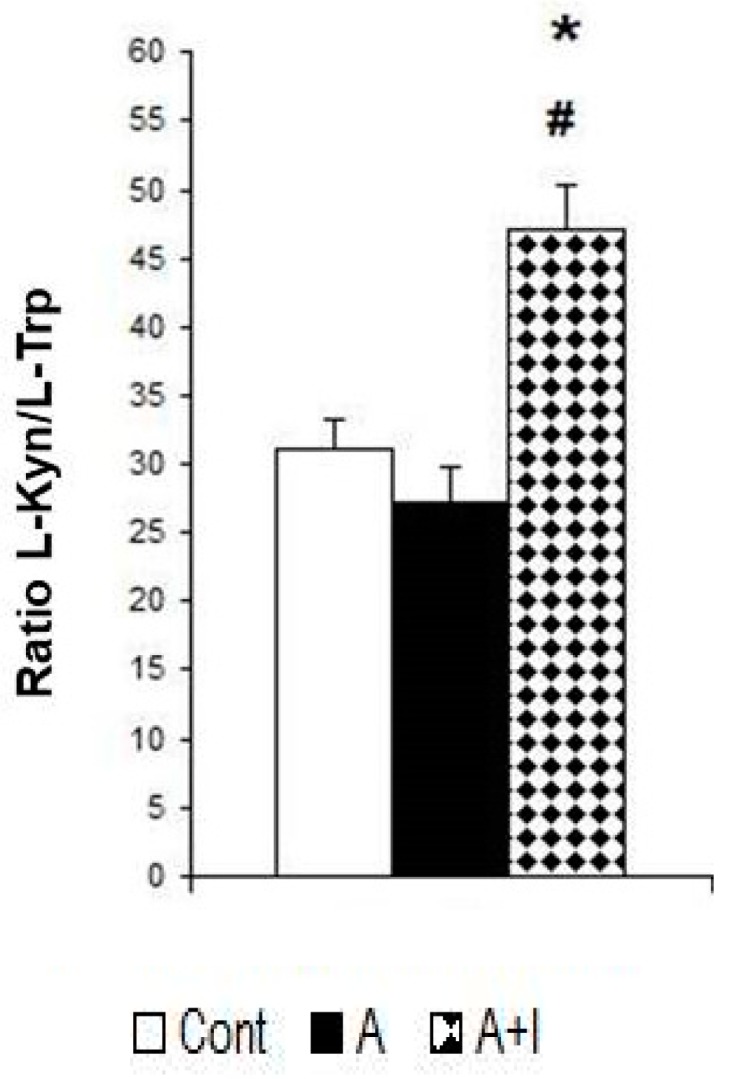
Patients with long-term titanium implants and amalgams have a higher L-kynurenine (Kyn)-L-trp ratio. **Cont**: control (*n* = 30). **Amalgam** (A, *n* = 55). **Dental titanium implants + Amalgam** (A + I, *n* = 57). * *p* < 0.05 vs. Control group (Cont), ^#^ *p* < 0.05 vs. Amalgam group (A).

Patients with long-term titanium dental implants and amalgams have a higher L-kynurenine/L-trp ratio than those with long-term dental amalgams alone [F (2, 140) = 14.63, *p* < 0.001]. However, this ratio is not affected in patients with long-term dental amalgams alone as compared to controls without dental biomaterials (*p* > 0.05, n.s). The r Spearman analysis indicated a strong correlation between systemic L-Kyn levels and the L-kyn/L-trp ratio (r = 0.78, *p* < 0.001, Figure 4 and Table 1).

Table 1 shows values for cytokines and chemokines plus relative error (standard deviation/n root) between experimental groups. The ANOVA data is shown by the F value and Kruskal–Wallis (KW) is associated with the H value. * *p* < 0.05 indicates a significant effect as compared to control. **^#^** *p* < 0.05 shows a significant effect as compared to patients with long-term dental amalgams alone (A). **Cont:** control (*n* = 30). **Amalgam** (A, *n* = 55). **Dental titanium implants + Amalgams** (A + I, *n* = 57).

### 3.5. Proinflammatory Mediators (Cytokines and Chemokines)

#### 3.5.1. IL-1 Beta Levels

**Figure 5:**
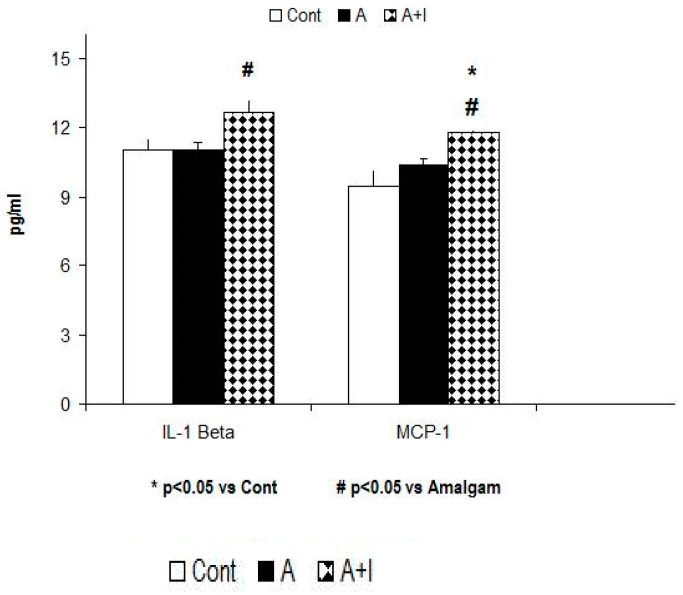
Increased IL-4 and MCP-1 levels in patients with long-term titanium dental implants and amalgams (A+I group) as compared to the A group (*p* < 0.05). **Cont:** control (*n* = 30). **Amalgam group** (A, *n* = 55). **Dental titanium + Amalgams** (A + I, *n* = 57). * *p* < 0.05 vs. Cont, ^#^ *p* < 0.05 vs. Amalgams (A).

The ANOVA data for systemic IL-1 Beta levels revealed a significant effect [F (2, 140) = 39.28; *p* < 0.01]. The Bonferroni post hoc revealed higher systemic IL-1 Beta levels for patients with long-term titanium dental implants and amalgams than those with long-term dental amalgams alone (*p* < 0.05). However, systemic levels did not differ between participants with dental amalgams alone and controls (*p* > 0.05, n.s, Figure 5).

#### 3.5.2. MCP-1 (CCL2)

The Kruskal–Wallis analysis revealed a significant effect on systemic MCP-1 levels (also called CCL-2) (H = 22.88, *p* < 0.001). Systemic MCP-1 is higher in patients who have long-term titanium dental implants and amalgams as compared with participants with long-term dental amalgams alone (*p* < 0.05). However, MCP-1 (CCL-2) levels did not differ between patients with long-term dental amalgams alone and controls (*p* > 0.05, n.s, Figure 5).

#### 3.5.3. IL-4 Anti-Inflammatory Cytokine

**Figure 6:**
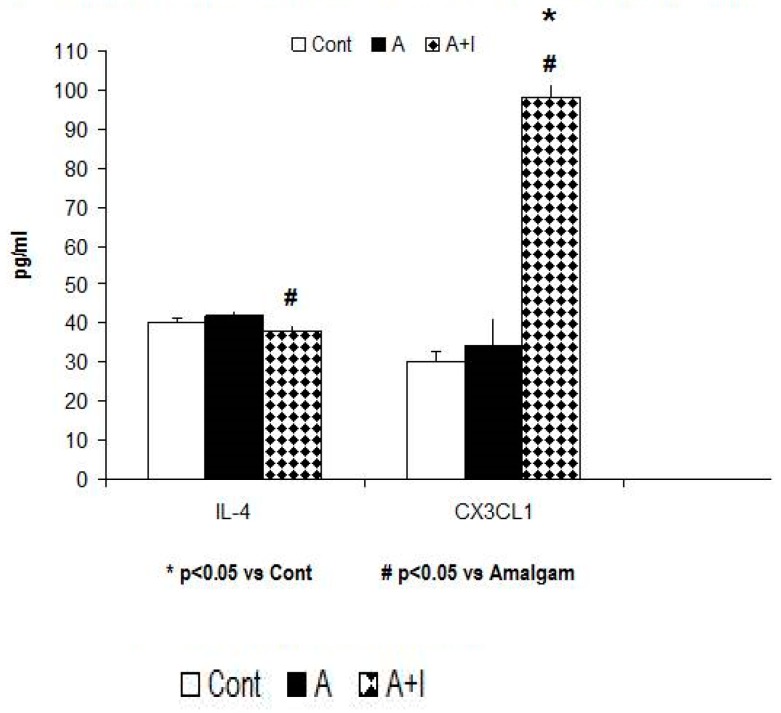
Patients with long-term titanium dental implants and amalgams have lower IL-4 levels as well as CX3CL1 (also called soluble fractalkine) elevations than in the A group. **Cont:** control (*n* = 30). **Amalgam** (A, *n* = 55). **Dental titanium implants + Amalgam** (A + I, *n* = 57). * *p* < 0.05 vs. Cont, ^#^ *p* < 0.05 vs. Amalgam (A).

The ANOVA data shows a slight rising tendency for this anti-inflammatory cytokine [F (2, 140) = 2.63; *p* = 0.082; n.s)]; the post hoc Bonferroni analysis reveals that IL-4 is greater in patients with long-term dental titanium implants and amalgams than in those with long-term dental amalgams alone (*p* < 0.05, Figure 6).

#### 3.5.4. Soluble Fractalkine (CX3CL1)

Soluble fractalkine (CX3CL1) levels were significantly higher in patients with long-term dental titanium implants and amalgams as compared to participants with long-term dental amalgams alone (*p* < 0.05). The ANOVA data revealed a systemic rise for this delta chemokine ligand [F (2, 140) = 5, *p* = 0.01]. The Bonferroni post hoc analysis indicated significantly higher soluble CX3CL1 levels in the A + I than in the A or Control groups (*p* < 0.05, Figure 6).

#### 3.5.5. Ratio IL-1 Beta/IL-4

**Figure 7:**
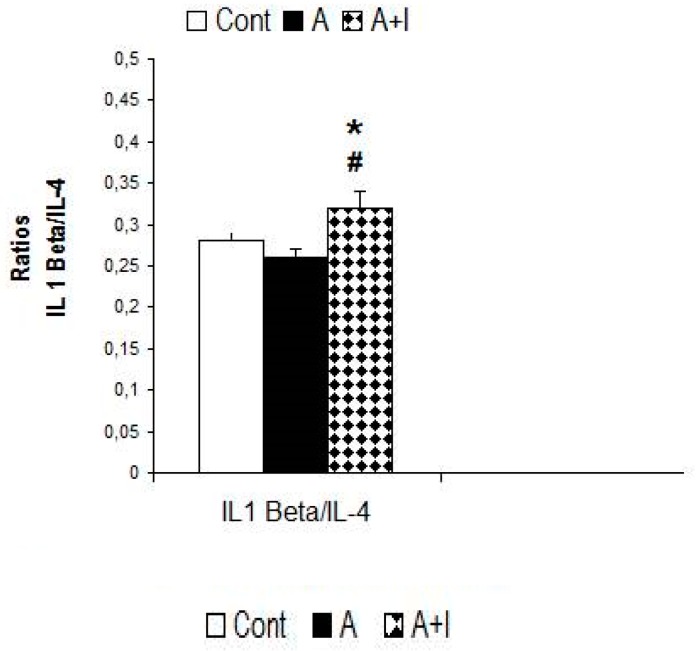
IL-1 Beta/IL-4 ratio in patients with long-term titanium implants and amalgams. **Cont**: control (*n* = 30). **Amalgam** (A, *n* = 55). **Dental titanium implants + Amalgams** (A + I, *n* = 57). * *p* < 0.05 vs. Cont, ^#^ *p* < 0.05 vs. Amalgam (A).

We found a slight tendency to higher levels in this ratio (H = 4.78, *p* = 0.091, n.s). The Mann–Whitney test revealed higher ratios in those with long-term dental amalgams and titanium implants than in participants with long-term dental amalgams alone (*p* < 0.05, Figure 7).

## 4. Discussion

The relatively high L-kyn/L-trp ratio observed here could signify a greater catabolism of L-Tryptophan (L-Trp) into L-kyn (kynurenine). The low L-tryptophan levels as well as elevations in CX3CL1 levels detected in A + I patients (with long-term dental titanium implants and amalgams) could predispose them to a silent chronic inflammation. Since the L-kyn/L-trp ratio is an indirect index of IDO activity [25], we suspect that lower systemic L-Trp levels may precede its catabolic conversion to kynurenine in patients with the long-term titanium dental implants. The rise in kynurenine found here (the first metabolite in the Trp catabolism) cannot be explained by intestinal malabsorption problems or oral carcinoma [32] since all the patients are healthy. Although IDO enzymatic activity was not evaluated, this elevated L-kyn/L-trp ratio in conjunction with elevations in IL-1 beta levels could indirectly reflect IDO activation [24,25] in the A + I group but not in the patients who only had long-term amalgam fillings. Although our patients are asymptomatic, their higher Il-1 Beta proinflammatory levels, as well as overactivation of the kynurenine pathway, could be important from a clinical viewpoint. A new role for L-trp metabolites has been proposed in bone metabolism. Apalset et al. demonstrated an association between kynurenine pathway metabolites and bone metabolism in patients since bone mineral density was associated with high serum concentrations of two interferon γ-induced kynurenines (xanthurenic acid, and 3-hydroxyanthranilic acid) [33].

Patients in the A + I group are older than those with long-term amalgams alone (A group). Chronic low-grade inflammation in the elderly is associated with an altered tryptophan and tyrosine (Tyr) metabolism [34] as also reflected in this study. However, these differences are not attributable to age differences between the study group with regard to systemic L-trp or Tyr levels.

The conversion of L-Trp (precursor) to melatonin not only promotes antioxidant and anti-inflammatory effects; this hormone also induces bone formation and resorption through several mechanisms [35,36,37,38]. Although melatonin levels were not evaluated in the present study, we would expect decreased melatonin concentrations to be concomitant to L-Trp reductions in patients with long-term dental titanium implants and amalgams. These resulting lower L-Trp levels could contribute to oxidative stress, leading to a chronic silent inflammatory state in the A + I group. Melatonin also decreases free radical production by regulating SuperOxide Dismutase (SOD-1) enzyme [38]. Applied topically, melatonin accelerates bone healing around dental implants in dogs [37,39,40]. A conversion of L-Trp into kyn could be essential in osteoblastogenesis and bone formation. In fact, the kynurenine pathway is an essential regulator of osteoblastogenesis and could make a potential new target for bone-forming cells in vivo [39]. Vidal et al. reported that mice without IDO activity are osteopenic; this feature suggests that elevations in kynurenine levels might indirectly reflect osteogenesis in A + I patients. Since melatonin attenuates titanium particle-induced osteolysis [36] and also stimulates dental implant osseointegration [37,40], this indirect evidence supports a role for kynurenine in osseointegration in patients with long-term dental titanium implants and amalgams.

Several studies reported toxicity of TiO_2_ nanoparticle in vitro as well as in rodent models [8,41]. The systemic IL-1 beta elevations observed in the A + I group concur with increased levels of this cytokine in endothelial cells exposed to TiO_2_ nanoparticles [17]. What is more, Il-1 Beta elevations have preceded implant failure in prospective studies [23]. However, there have been no implant failures or systemic titanium elevations in our cohort (data not shown), concurring with a study reporting that arthroplasty patients had no systemic Ti elevations [42].

The cobalt/chromium alloy found in the implant crowns is more resistant to corrosion that other biomaterials; it exhibits high elasticity and minimizes allergic reactions with surrounding tissues [43]. However, we observed oxidative stress and elevated systemic lipoperoxides in patients with long-term dental titanium implants as well as higher Co in the A + I group than in the A group [44]. Since Co induces inflammatory responses in macrophages in vitro [44,45,46], the possibility that Co ion release may promote inflammation should not be ignored in the A + I group. In fact, Co^2+^ increases the secretion of interleukin-8 (IL-8), CXCL10 and MCP-1 = CCL-2 chemotactic chemokines in MonoMac 6 macrophages [44]. In addition, conditioned media from Co-stimulated cells showed increased chemotaxis in response to neutrophils in vitro [44,45,46], a classical function of chemokines [27]. However, a direct relationship between elevated Co levels and chemokines cannot be determined in our patients (A + I group). Nevertheless, our indirect findings suggest enhanced chemotaxis in patients with long-term dental amalgams as a consequence of a silent chronic inflammatory state. The inflammation could increase the catabolic degradation of L-Trp into kyn in patients with long-term dental titanium implants and amalgams (A + I group). The activation of the kynurenine pathway plays an important role in osteoblastogenesis in vitro, and this process can be accelerated by the exogenous addition of proinflammatory mediators like IFN γ [39].

Their higher kynurenine levels may raise soluble fractalkine (CX3CL1) and MCP-1 chemokine levels in the A + I group; this possibility suggests enhanced chemotactic activity in these patients (A + I group). In fact, excitotoxic conditions could raise CX3CL1 (or soluble fractalkine) levels [41] and the catabolic subproducts of L-Trp are also excitotoxic mediators [47]. In agreement with the elevated systemic CX3CL1 levels found in our study, Lechner et al. 2018 reported increased RANTES chemokine levels (regulated on activation by normal T cell expression also known as Chemokine C–C motif ligand 5, CCL5) in patients with titanium implants [48].

Taylor and Feng have demonstrated the association between proinflammatory interferon γ, indoleamine 2,3-dioxygenase and the tryptophan catabolism [25]. The CX3CL1 chemokine ligand promotes pleiotropic effects by activating CX3CR1 in blood and other cell types [28]. Consistent with the IDO-induced Trp metabolism suggested in our study, the ratio of 3-hydroxykynurenine (3-HK) to Trp was increased in the brain 2 h after LPS: lipopolysaccharide treatment (a systemic inductor of inflammation) in transgenic mice lacking the CX3CR1 chemokine receptor (CX3CR1-/-). Increased serotonin (5-HT) turnover was also evident in the mice brain. The LPS (a systemic inductor of inflammation) associated increases in both the 3-HK:Trp and 5-HIAA:5-HT ratios were prevented by the inhibition of IDO in these mice [29,49]. Moreover, IL-1β and CD14 elevated expression was still detected in the microglia of CX3CR1-/- knock out (the chemokine receptor for CX3CL1 ligand) mice 24 h after an LPS i.p (intraperitoneal injection) injection [29]. These indirect findings also suggest that kynurenine and CX3CL1 release could contribute to a silent chronic inflammation in patients with long-term dental implants and amalgams. On the basis of this evidence, we really should consider the possibility that both CX3CL1 and MCP-1 chemokines promote chemotactic responses in patients with long-term dental titanium implants and amalgams.

Vallés et al. demonstrated that primary macrophages released greater amounts of Tumor Necrosis factor (TNF-alpha), IL-6 and IL-1beta proinflammatory mediators after incubation with titanium particles [50]. While proinflammatory cytokines like TNF-α and IL-6 are distributed early on during the acute stage of inflammation, other chemokines like RANTES or CX3CL1 may be activated at a later time; these latter chemokines play a crucial role in the transition of acute inflammation into a more chronic inflammatory phenomenon [47]. Proinflammatory signaling induced by RANTES or CX3CL1 chemokine ligands in particular systemically affect the organism [51] and may result in a chronic inflammatory state. Thus, soluble fractalkine (CX3CL1) signaling may contribute to the development of chronic inflammation similarly to the way in which systemic RANTES induced medullar osteonecrotic changes in the jawbone of patients with titanium implants [48]. An elevation in CX3CL1 levels could provoke a transition from an acute to a chronic inflammatory state in patients with long-term dental implants and amalgams.

In addition, patients with long-term dental titanium and amalgams are more susceptible to oxidative stress [52]; thus, altered levels of certain amino acids like L-Trp could reflect systemic metabolic imbalances [53,54,55,56]. What is more, fractalkine receptor (CX3CR1) deficiency sensitizes mice and increases the turnover of tryptophan, serotonin, and dopamine in the brain 24 h after an i.p LPS injection in knockout CX3CL1-/- transgenic mice, even though these increases were independent of CX3CR1 expression [29]. Collectively, a relationship between L-kyn, CX3CL1 and MCP-1 chemokine levels can be suspected in patients with long-term dental titanium implants and amalgams.

## 5. Conclusions

The IL-1 Beta elevations observed in patients with long-term dental implants and amalgams, despite having titanium levels comparable to the controls, could reflect a chronic systemic inflammatory response. In fact, patients with long-term titanium implants and dental amalgams (A + I) have higher systemic L-kyn/L-trp ratios concomitant to L-Trp reduction, something that could indirectly reflect osseointegration. Higher L-Kynurenine levels could reflect catabolic L-Trp degradation as a consequence of IL-1 Beta, MCP-1 and CX3CL1 systemic release in the A + I group as compared to those with long-term dental amalgams alone (A). Clinical trials are needed to confirm the predictive role of CX3CL1, L-Kyn/L-trp and IL-1 Beta levels as systemic inflammatory biomarkers associated with bone-osseointegration in patients with long-term titanium dental implants; a higher CX3CL1 and MCP-1 chemokine production would reflect enhanced chemotactic responses in these patients (A + I group).

## Figures and Tables

**Table 1:** Systemic cytokine levels and L-kyn/L-trp ratios.

	Cont	Amalgam (A)	Amalgam + Implants (A + I)	KW
IL-1 β (pg/mL)	11.03 ± 0.48	11.03 ± 0.32	12.66 ± 0.52 ^#^	H = 9.37, *p* = 0.009
MCP-1 (pg/mL)	9.48 ± 0.7	10.39 ± 0.24	11.76 ± 0.14 *^,#^	H = 22.88, *p* < 0.001
CX3CL1 (pg/mL)	30.02 ± 2.7	34.3 ± 7.1	98 ± 3.6 *^,#^	F (2, 140) = 5, *p* = 0.01
IL-4 (pg/mL)	40 ± 1.3	42 ± 1.1	37.8 ± 1.7 ^#^	F (2, 140) = 2.63, *p* = 0.082
IL 1 Beta/Il-4 ratio	0.28 ± 0.01	0.26 ± 0.011	0.32 ± 0.02 ^#^	H (4, 140), *p* = 0.091, n.s
Kyn	1.47 ± 0.09	1.68 ± 0.1	2.09 ± 0.14 *^,#^	F (2, 140) = 5.96, *p* = 0.035
Kyn/L-Trp ratio	31 ± 2.3	27 ± 2.5	47 ± 3.3 *^,#^	F (2, 140) = 14.6, *p* < 0.001

* *p* < 0.05 vs. Cont, # *p* < 0.05 vs. Amalgam (A).

## References

[1] Cabaña-Muñoz M.E., Parmigiani-Izquierdo J.M., Bravo-González L.A., Kyung H.M., Merino J.J. (2015). Increased Zn/glutathione levels and higher superoxide dismutase-1 activity as biomarkers of oxidative stress in women with long-term dental amalgam fillings: correlation between mercury/aluminium levels (in Hair) and antioxidant systems in plasma. PLoS ONE.

[2] Shraim A., Alsuhaimi A., Al-Thakafy J.T. (2011). Dental clinics: A point pollution source, not only of mercury but also of other amalgam constituents. Chemosphere.

[3] Bishara S.E., Barrett R.D., Selim M.I. (1993). Biodegradation of orthodontic appliances. Part II. Changes in the blood level of nickel. Am. J. Orthod. Dentofac. Orthop..

[4] Puchyr R.F., Bass D.A., Gajewski R., Calvin M., Marquardt W., Urek K., Druyan M.E., Quig D. (1998). Preparation of hair for measurement of elements by inductively coupled plasma-mass spectrometry (ICP-MS). Biol. Trace Elem. Res..

[5] Merino J.J., Arce C., Naddaf A., Bellver-Landete V., Oset-Gasque M.J., González M.P. (2014). The nitric oxide donor SNAP-induced amino acid neurotransmitter release in cortical neurons. Effects of blockers of voltage-dependent sodium and calcium channels. PLoS ONE.

[6] Mutter J. (2011). Is dental amalgam safe for humans? The opinion of the scientific committee of the European Commission. J. Occup. Med. Toxicol..

[7] Park J.D., Zheng W. (2012). Human exposure and health effects of inorganic and elemental mercury. J. Prev. Med. Public Health.

[8] Lai J.C., Lai M.B., Jandhyam S., Dukhande V.V., Bhushan A., Daniels C.K., Leung S.W. (2008). Exposure to titanium dioxide and other metallic oxide nanoparticles induces cytotoxicity on human neural cells and fibroblasts. Int. J. Nanomed..

[9] Lim S.D., Takada Y., Kim K.M., Okuno O. (2003). Ions released from dental amalgams in contact with titanium. Dent. Mater. J..

[10] Barrett R.D., Bishara S.E., Quinn J.K. (1993). Biodegradation of orthodontic appliances. Part, I. Biodegradation of nickel and chromium in vitro. Am. J. Orthod. Dentofac. Orthop..

[11] Joska L., Fojt J., Cvrcek L., Brezina V. (2014). Properties of titanium-alloyed DLC layers for medical applications. Biomatter.

[12] Huerta-García E., Pérez-Arizti J.A., Márquez-Ramírez S.G., Delgado-Buenrostro N.L., Chirino Y.I., Iglesias G.G., López-Marure R. (2014). Titanium dioxide nanoparticles induce strong oxidative stress and mitochondrial damage in glial cells. Free Radic. Biol. Med..

[13] Feng X., Chen Y., Zhang J., Wang J., Shao L., Wei L. (2015). Application of dental nanomaterials: Potential toxicity to the central nervous system. Int. J. Nanomed..

[14] Shi H., Magaye R., Castranova V., Zhao J. (2013). Titanium dioxide nanoparticles: A review of current toxicological data. Part. Fibre Toxicol..

[15] Shimizu M., Tainaka H., Oba T., Mizuo K., Umezawa M., Takeda K. (2009). Maternal exposure to nanoparticulate titanium dioxide during the prenatal period alters gene expression related to brain development in the mouse. Part. Fibre Toxicol..

[16] Olmedo D.G., Tasat D., Guglielmotti M.B., Cabrini R.L. (2003). Titanium transport through the blood stream. An experimental study on rats. J. Mater. Sci. Mater. Med..

[17] Coccini T., Grandi S., Lonati D., Locatelli C., De Simone U. (2015). Comparative cellular toxicity of titanium dioxide nanoparticles on human astrocyte and neuronal cells after acute and prolonged exposure. Neurotoxicology.

[18] Van Donkelaar E.L., Blokland A., Ferrington L., Kelly P.A.T., Steinbusch H.W.M., Prickaerts J. (2011). Mechanism of acute tryptophan depletion: Is it only serotonin?. Mol. Psychiatry.

[19] Young S.N., Smith S.E., Pih R.O., Ervin F.R. (1985). Tryptophan depletion causes a rapid lowering of mood in normal males. Psychopharmacology.

[20] Permuy M., López-Peña M., González-Cantalapiedra A., Muñoz F. (2017). Melatonin: A review of its potential functions and effects on dental diseases. Int. J. Mol. Sci..

[21] Jacobi-Gresser E., Huesker K., Schütt S. (2013). Genetic and immunological markers predict titanium implant failure: A retrospective study. Int. J. Oral Maxillofac. Surg..

[22] Catena-Dell’Osso M., Rotella F., Dell’Osso A., Fagiolini A., Marazziti D. (2013). Inflammation, serotonin and major depression. Curr. Drug Targets.

[23] Oxenkrug G.F. (2010). Tryptophan kynurenine metabolism as a common mediator of genetic and environmental impacts in major depression disorders: The serotonin hypothesis revised 40 years later. Isr. J. Psychiatry.

[24] Mandi Y., Vecsei L. (2012). The kynurenine system and immunoregulation. J. Neural Trasm..

[25] Taylor M.W., Feng G.S. (1991). Relationship between interferon-gamma, indoleamine 2,3-dioxygenase, and trypthophane catabolism. FASEB J..

[26] Fallarino F., Grohmann U., Pucceti P. (2012). Indoleamine 2,3-dioxygenase: From catalysis to signaling function. Eur. J. Immunol..

[27] Deshmane S.L., Kremlev S., Amini S., Sawaya B.E. (2009). Monocyte chemoattractant protein-1 (MCP-1): An overview. J. Interferon Cytokine Res..

[28] Chapman G.A., Moores K.E., Gohil J., Berkhout T.A., Patel L., Green P., Macphee C.H., Stewart B.R. (2000). The role of fractalkine in the recruitment of monocytes to the endothelium. Eur. J. Pharm..

[29] Corona A.W., Huang Y., O’Connor J.C., Dantzer R., Kelley K.W., Popovich P.G., Godbout J.P. (2010). Fractalkine receptor (CX3CR1) deficiency sensitized mice to the behaviour changes induced by lipopolysaccharide. J. Neuroinflamm..

[30] Cabaña-Muñoz M.E., Parmigiani-Izquierdo J.M., Parmigiani-Cabaña J.M., Merino J.J. (2015). Safe removal of amalgam fillings in dental clinic: Use of synergic nasal filters (active carbon) and phytonaturals. Int. J. Sci. Res. (IJSR).

[31] Merino J., Aller M.A., Rubio S., Arias N., Nava M.P., Loscertales M., Arias J., Arias J.L. (2011). Gut-brain chemokine changes in portal hypertensive rats. Dig. Dis. Sci..

[32] Apalset E.M., Gjesdal C.G., Ueland P.M., Midttun Ø., Ulvik A., Eide G.E., Meyer K., Tell G.S. (2014). Interferon (IFN)-γ-mediated inflammation and the kynurenine pathway in relation to bone mineral density: The Hordaland Health Study. Clin. Exp. Immunol..

[33] Wang S.H., Chang C.W., Chou H.C. (2015). Methoxytryptophan-dependent inhibition of oral squamous cell carcinoma metastasis. Electrophoresis.

[34] Capuron L., Schroecksnadel S., Féart C., Aubert A., Higueret D., Barberger-Gateau P., Layé S., Fuchs D. (2011). Chronic low-grade inflammation in elderly persons is associated with altered tryptophan and tyrosine metabolism: Role in neuropsychiatric symptoms. Biol. Psychiatry.

[35] Tresguerres I.F., Clemente C., Blanco L., Khraisat A., Tamimi F., Tresguerres J.A. (2012). Effects of local melatonin application on implant osseointegration. Clin. Implant Dent. Relat. Res..

[36] Ping Z., Hu X., Wang L., Shi J., Tao Y., Wu X., Hou Z., Guo X., Zhang W., Yang H. (2017). Melatonin attenuates titanium particle-induced osteolysis via activation of Wnt/β-catenin signaling pathway. Acta Biomater..

[37] Cutando A., Gómez-Moreno G., Arana C., Muñoz F., Lopez-Peña M., Stephenson J., Reiter R.J. (2008). Melatonin stimulates osteointegration of dental implants. J. Pineal Res..

[38] Cutando A., Arana C., Gómez-Moreno G., Escaes G., López A., Ferrera M.J., Reiter R.J., Acuña-Castroviejo D. (2007). Local application of melatonin into alveolar sockets of beagle dogs reduces tooth-removal oxidative stress. J. Periodontol..

[39] Vidal C., Li W., Santner-Nanan B., Lim C.K., Guillemin G.J., Ball H.J., Hunt N.H., Nanan R., Duque G. (2015). The kynurenine pathway of tryptophan degradation is activated during osteoblastogenesis. Stem Cells.

[40] Palin L.P., Polo T.O.B., Batista F.R.S., Gomes-Ferreira P.H.S., Garcia Junior I.R., Rossi A.C., Freire A., Faverani L.P., Sumida D.H., Okamoto R. (2018). Daily melatonin administration improves osseointegration in pinealectomized rats. J. Appl. Oral Sci..

[41] Mohammadipour A., Fazel A., Haghir H., Motejaded F., Rafatpanah H., Zabihi H., Hosseini M., Bideskan A.E. (2014). Maternal exposure to titanium dioxide nanoparticles during pregnancy, impaired memory and decreased hippocampal cell proliferation in rat offspring. Environ. Toxicol. Pharm..

[42] Geraets L., Oomen A.G., Krystek P., Jacobsen N.R., Wallin H., Laurentie M., Verharen H.W., Brandon E.F., de Jong W.H. (2014). Tissue distribution and elimination after oral and intravenous administration of different titanium dioxide nanoparticles in rats. Part. Fibre Toxicol..

[43] Anjum S.A., Lawrence H., Holland J.P., Kirby J.A., Deehan D.J., Tyson A.J. (2016). Effect of cobalt-mediated Toll-like receptor 4 activation on inflammatory responses in endothelial cells. Oncotarget.

[44] Cabaña-Muñoz M.E., Parmigiani-Izquierdo J.M., Camacho-Alonso F., Merino J.J. (2019). Increased Systemic Malondialdehyde Levels and Decreased Mo/Co, Co/Fe2+ Ratios in Patients with Long-Term Dental Titanium Implants and Amalgams. J. Clin. Med..

[45] Eliaz N. (2019). Corrosion of metallic biomaterials: A review. Materials.

[46] Ghallab N.A., Hamdy E., Shaker O.G. (2016). Malondialdehyde, superoxide dismutase and melatonin levels in gingival crevicular fluid of aggressive and chronic periodontitis patients. Aust. Dent. J..

[47] Lawrence H., Deehan D., Holland J., Kirby J., Tyson-Capper A. (2014). The immunobiology of cobalt: Demonstration of a potential aetiology for inflammatory pseudotumours after metal-on-metal replacement of the hip. Bone Jt. J..

[48] Lechner J., Noumbissi S., von Baehr V. (2018). Titanium implants and silent inflammation in jawbone-a critical interplay of dissolved titanium particles and cytokines TNF-α and RANTES/CCL5 on overall health?. EPMA J..

[49] Corona A.W., Norden D.M., Skendelas J.P., Huang Y., O’Connor J.C., Lawson M., Dantzer R., Kelly K., Godbout J.P. (2013). Indoleamine 2,3-dioxygenase inhibition attenuates lipopolysaccharide induced persistent microglial activation and depressive-like complications in fractalkine receptor (CX3CR1)-deficient mice. Brain Behav. Immun..

[50] Vallés G., González-Melendi P., González-Carrasco J.L., Saldaña L., Sánchez-Sabaté E., Munuera L., Villaboa N. (2006). Differential inflammatory macrophage response to rutile and titanium particles. Biomaterials.

[51] Lechner J., von Baehr V. (2013). RANTES and fibroblast growth factor 2 in jawbone cavitations: Triggers for systemic disease?. Int. J. Gen. Med..

[52] Merino J.J., Parmigiani-Izquierdo J.M., Toledano Gasca A., Cabaña-Muñoz M.E. (2019). The Long-Term Algae Extract (*Chlorella and Fucus sp*) and Aminosulphurate Supplementation Modulate SOD-1 Activity and Decrease Heavy Metals (Hg^++^, Sn) Levels in Patients with Long-Term Dental Titanium Implants and Amalgam Fillings Restorations. Antioxidants.

[53] Gostner J.M., Geiser S., Stoning M., Mair L., Spencer-Unterweger B., Fuchs D. (2019). Tryptophan metabolism and related pathways in psychoneuroimmunology: The impact of nutrition and lifestyle. Neuropsychobiology.

[54] Sas K., Robotka H., Toldi J., Vécsei L. (2007). Mitochondria, metabolic disturbances, oxidative stress and the kynurenin system, with focus on neurodegenerative disorsers. J. Neurol. Sci..

[55] Blankfield A. (2013). Kynurenine Pathway Pathologies: Do Nicotinamide and Other Pathway Co-Factors have a Therapeutic Role in Reduction of Symptom Severity, Including Chronic Fatigue Syndrome (CFS) and Fibromyalgia (FM). Int. J. Tryptophan Res..

[56] Bortolato B., Berk M., Maes M., McIntyre R.S., Carvalho A.F. (2016). Fibromyalgia and bipolar disorder: Emerging epidemiological associations and shared pathophysiology. Curr. Mol. Med..

